# YABBY3-Orthologous Genes in Wild Tomato Species: Structure, Variability, and Expression

**Published:** 2017

**Authors:** M. A. Filyushin, M. A. Slugina, A. V. Shchennikova, E. Z. Kochieva

**Affiliations:** Federal State Institution «Federal Research Centre «Fundamentals of Biotechnology» of the Russian Academy of Sciences», Leninsky pr. 33, bldg. 2, Moscow, 119071, Russia; Department of Biotechnology, Faculty of Biology, Moscow State University, Leninskie Gory 1, bldg. 12, Moscow, 119991, Russia

**Keywords:** YABBY3, polymorphism, qRT-PCR, Solanum section Lycopersicon, adaxial-abaxial asymmetry

## Abstract

Evolution of the genes encoding YABBY transcription factors is believed to be one of the key reasons for flat leaf emergence from the radially symmetrical stem and gynoecium diversity. *YABBY *genes determine the identity of the abaxial surface of all aboveground lateral organs in seed plants. In the present study, complete sequences of *YABBY3-*orthologous genes were identified and characterized in 13 accessions of cultivated and wild tomato species with diverse morphophysiology of leaves, flowers, and fruits. The obtained gene sequences showed high homology (95–99%) and an identical exon-intron structure with the known *S. lycopersicum YABBY3 *gene, and they contained sequences that encode the conserved HMG-like YABBY and Cys2Cys2-zinc-finger domains. In total, in the analyzed *YABBY3 *genes, 317 variable sites were found, wherein 8 of 24 exon-specific SNPs were nonsynonymous. In the vegetative and reproductive organs of red-fruited and green-fruited tomato species, *YABBY3 *gene expression was similar to that in *S. pimpinellifolium *described earlier, but it demonstrated interspecies differences at the leaf-, bud- and flower-specific expression levels.

## INTRODUCTION

Plant growth and development processes are controlled by transcription factors, whose evolution is one of the major causes of morphological diversity in the plant kingdom [1-4]. The origin of the flower and reproductive organs is believed to be related to the duplication and changes in MADS-box transcription factor genes [5, 6]. At the same time, flat leaf emergence from the radially symmetrical stem, as well as gynoecium diversity, is considered to be a consequence of YABBY transcription factor genes evolution [7]. The presence of these genes in angiosperm and gymnosperm plants and their absence in moss and lycopodium [8-10] suggest that *YABBY *genes originate from one or two predecessors in the last common ancestor of seed plants [10-12]. Diversification of *YABBY *genes led to the occurrence of individual family members with unique roles in leaf, carpel, and ovule development [8, 11, 13, 14], including the *YABBY2 *and *YABBY5 *genes*, *which were presumably involved in the evolutionary divergency of the pistil stalk and stamen filament morphology [15, 16]. Other *YABBY *gene families, *INNER NO OUTER (INO) *and *CRABS CLAW (CRC), *apparently developed in parallel with the evolution of the carpel and ovule during leaf-like reproductive sporophyll modification [11, 17].

In dicots and monocots, *YABBY *genes play similar roles in leaf and leaf-like organs development, specifying their abaxial-adaxial asymmetry and lamina growth, as well as leaf boundaries [4, 10, 18]. Additionally, *YABBY *genes are involved in the formation of such flower organs as nectaries, carpels, etc. [19-21]. To date, the functions of certain YABBY proteins have only been described in the model plant *Arabidopsis thaliana*. Thus, it has been shown that YABBY1 (syn. FILAMENTOUS FLOWER, FIL), YABBY3, and YABBY5, along with other components of the transcription complex, support the identity of abaxial leaf surface cells and are also involved in the initiation of embryonic shoot apical meristem and its postembryonal maintenance [22]. Activation of a certain *YABBY *gene expression in the nectaries and carpels involves MADS-domain proteins [23]. In turn, YABBY1, together with other transcription factors, controls the spatial activity of MADS-box genes and, thus, is involved in floral organ primordia initiation in the correct position and number, determining the corresponding cell’s fate [24-26].

*YABBY *genes encode small proteins (180–250 amino acid residues) containing two conserved domains [27, 28]. The N-terminal part of the protein includes the Cys2Cys2-zinc-finger motif, and the C-terminus includes the YABBY domain.

In plant genomes, the *YABBY *genes number differs. In *A. thaliana*, six *YABBY *genes were found; four of them (*YABBY1, YABBY2*, *YABBY3, *and *YABBY5) *are mainly expressed in leaves and leaf-like organs (cotyledons, sepals, petals, stamens, and carpels), while the other two *(CRC *and *INO) *are expressed in some parts of floral reproductive organs [10, 23, 27]. Eight genes were identified in rice *Oryza sativa*; moreover, each *OsYABBY2* and *OsYABBY7 *has two alternatively spliced transcripts [29].

Nine *YABBY *genes (*YABBY1*, *YABBY2*, *YABBY3*, *YABBY5a*, *YABBY5b*, *CRCa*, *CRCb*, *FAS*, and *INO*) were identified in cultivated tomato *(Solanum lycopersicum), *which is one of the major vegetable crops [30, 31]. *S. lycopersicum, *along with 12 wild related species, comprises the Lycopersicon section of the *Solanum *genus [32]. Tomato species widely vary in their morphophysiological characteristics, including leaf and flower morphology. Depending on the mating system structure, tomatoes are divided into self-compatible and self-incompatible species. The latter are characterized by high polymorphism, large flowers, and exerted stigma [32]. It is known that the plant reproductive system, which depends on the flower’s morphophysiology, as well as the differences in the leaf structure, can result from the different activities of YABBY transcription factors [7]. First and foremost, this relates to YABBY1/ YABBY3 proteins, which are expressed in almost all asymmetric aboveground plant organs.

The present study was focused on the identification of *YABBY3*-orthologous genes in wild tomato species and an evaluation of their polymorphism. To date, complete *YABBY3 *sequences are determined only for two tomato species: *S. lycopersicum *and *S. pennellii*, and *YABBY3 *expression patterns were characterized only in *S. lycopersicum *[31] and *S. pimpinellifolium *[30]. Therefore, the present results, based on an analysis of a large number of tomato species, will contribute to our knowledge of *YABBY *genes and their possible functions.

## EXPERIMENTAL

A set of 13 accessions of 11 tomato species from the collection of the All-Russian Institute of Plant Genetic Resources n.a. N.I. Vavilov (VIR) was selected for this study. The analyzed species differed both in the mating system and fruit morphology (*Table 1*).

**Table 1:** The cultivated and wild tomato species used in the present study

Species/subspecies/cultivar	VIR Ref. No	Crossing system	Color of the ripe fruit
S. cheesmaniae (Riley) Fosberg	3969	self-compatible	Red
S. galapagense Darwin & Peralta	3970	self-compatible	Red
S. lycopersicum var. humboldtii (Willd.) Dunal	2912	self-compatible	Red
S. lycopersicum L., cv. Silvestre recordo	1580	self-compatible	Red
S. pimpinellifolium var. racemigerum (Lange) Brezhnev	1018	self-compatible	Red
S. chmielewskii (Rick, Kesicki, Fobes & Holle) Spooner, Anderson & Jansen	13725	self-compatible	Green
S. neorickii Spooner, Anderson & Jansen	5033	self-compatible	Green
S. arcanum Peralta	13958	self-incompatible	Green
S. chilense ( Dunal) Reiche	4300	self-incompatible	Green
S. corneliomulleri Macbr.	4367	self-incompatible	Green
S. habrochaites Knapp & Spooner	13964	self-incompatible	Green
S. peruvianum L.	4361	self-incompatible	Green
S. peruvianum var. dentatum (Dunal) Dunal	3966	self-incompatible	Green

The plants were grown from seeds in a greenhouse (8/16 h night/day; 23/28°C night/day, light intensity 300–400 mM/m2). Genomic DNA was isolated from leaves using ZR-96 Plant/Seed DNA Kit (Zymo research, Irvine, USA). Five weeks after planting in the greenhouse, as fruit formation started, tissue samples were collected simultaneously from each plant, including leaves, young buds, open flowers, and immature green fruits, at 9.00-12.00 a.m. The sampled material was immediately frozen and ground in liquid nitrogen. Total RNA was isolated using a RNeasy Plant Mini Kit (QIAGEN, Hilden, Germany) and used for cDNA synthesis with a GoScript kit (Promega, Madison, USA).

Specific primers, sYB3F (5’-AATCAAATCAATCACAAAARCAG-3’) and sYB3R (5’-CACATTAATTGGTTAGACACTTA-3’), were designed based on the complete *YABBY3 *gene sequence of *S. lycopersicum *(GeneID: 101247051) and *S. pennellii *(GeneID: 107026918) for an amplification of the full-length copies of this gene in the examined species*. *Additional internal primers, sYB3ex2R (5’-ATTAGTGCAGTGTCCACATC-3’) and sYB3ex4R (5’-TTGATGAATCGGTTGTAAGC-3’), were designed for sequencing. The genes were amplified using LongAmp® polymerase Hot Start Taq DNA Polymerase (USA) under the following conditions: initial denaturation (10 min, 94°C); 35 cycles of denaturation (30 sec, 94°C); annealing (30 sec, 58°C) and elongation (4 min, 65°C); and final elongation (10 min, 65°C). PCR fragments were purified using a QIAEX® II Gel Extraction kit (QIAGEN, Hilden, Germany), cloned into the plasmid vector pGEMT-easy (Promega, Madison, USA), and sequenced using the BigDye system and an Applied Biosystems 3730 DNA analyzer (Applied Biosystems, Waltham, United States; Core Facility “Bioengineering”).

The obtained sequences were aligned and analyzed using the MEGA 7.0 [33]. The comparative analysis was carried out using known *YABBY3 *complete sequences of two tomato species, *S. lycopersicum *cv. Heinz (GeneID:101247051) and *S. pennellii *(GeneID: 107026918), potato *S. tuberosum *(GeneID: 102577797), and *A. thaliana *(GeneID : 827 914). The positions of nucleotide and amino acid substitutions were determined in comparison with the *S. lycopersicum *cv. Heinz *YABBY3 *(GeneID: 101247051). The structural domains of YABBY3 orthologs were determined using NCBI-CDD (http://www.ncbi.nlm.nih.gov/Structure/cdd/wrpsb. cgi) and published data [27, 28]. Known sequences of YABBY genes, cDNAs, and the proteins of *S. lycopersicum *(*SlYABBY1 *(XM_004229745), *SlYABBY2 *(XM_004241308), *SlYABBY3 *(XM_004245689), *SlYABBY5a *(XM_004242730), *SlYABBY5b *(XM_004251674), *SlFAS *(NM_001247461), *SlINO *(XM_004239291), *SlCRCa *(XM_004238984), *SlCRCb *(XM_004228801)), and *A. thaliana *(*AtYABBY1 *(AF136538), *AtYABBY2 *(AF136539), *AtYABBY3 *(AF136540), *AtYABBY5 *(NM_179750), *AtINO *(AF195047), *AtCRC *(AF132606)) were subjected to a phylogenetic analysis performed using the MEGA 7.0 Maximum Likelihood method (ML), preassigned by the Modeltest program. The possible effects of the amino acid substitutions on the protein structure and function were assessed using the Grantham matrix [34] and PROVEAN [35]. The three-dimensional protein structure was analyzed using the Phyre2 program [36] and visualized by Chimera 1.11.2 (http://www.cgl.ucsf.edu/chimera/).

*YABBY3*-orthologous genes expression was determined in young leaves, young buds, open flowers, and green immature fruits by quantitative real-time PCR (qRT-PCR) using the Reaction mixture for qRT-PCR in the presence of SYBR GreenI and a ROX kit (Syntol, Moscow, Russia) on a CFX96 Real-Time PCR Detection System (Bio-Rad Laboratories, Hercules, USA). qRT-PCR was carried out using a gene-specific primer pair: tY3rt1F (5’-GTCACACTTACTTCTCTCCTTCAC-3’) and tY3rtR (5’-CAGGAGGTCTGTTAACAACGG-3’). The reactions were carried out in two biological and three technical replicates under the following conditions: 95°C – 5 min; 40 cycles (95°C – 15 sec, 62°C – 50 sec). The relative expression level was assessed using the *CAC *gene as a reference [37]. The statistical analysis was performed using GraphPad Prism v. 7.02, including the assessment of the statistical significance of the expression differences in various organs of each analyzed tomato species using the unpaired t-test with Welch’s correction (*Table 3*).

## RESULTS AND DISCUSSION

**Fig. 1:**
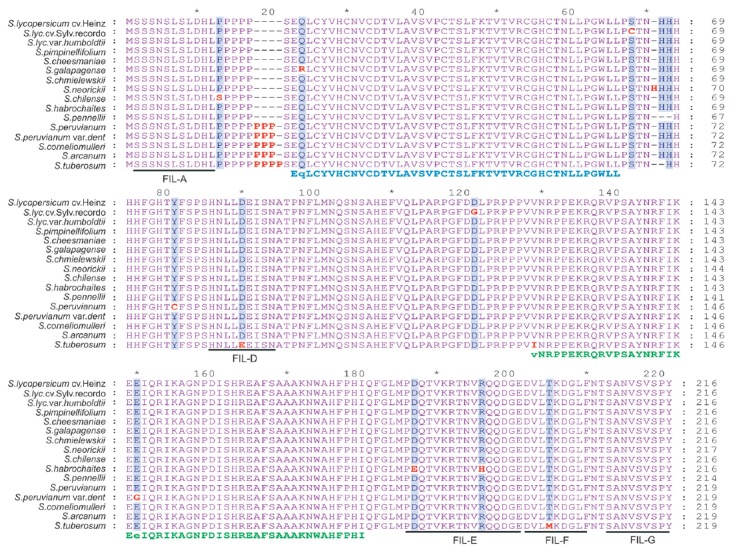
Alignment of YABBY3 amino acid sequences from accessions of tomato and potato (*S. tuberosum*) species. The Zinc-finger and YABBY domains are indicated by blue and green letters, respectively, under alignment. Indels and substitutions are highlighted in red. Conserved motifs specific to YABBY1/YABBY3 clade are underlined and named

Complete sequences of the *YABBY3*-orthologous genes were determined in 13 accessions of 11 tomato species (*Solanum* section Lycopersicon). The comparative analysis of these sequences showed that they are highly homologous (95–99% similarity) to the known tomato *YABBY3 *gene (ID: 101247051). The total length of the gene varied from 2622 bp in *S. neorickii *to 2713 bp in *S. cheesmaniae*. The genes were composed of seven exons and six introns (*Table 2*) and included sequences that encoded the conserved HMG-like YABBY (125–176 aa) and the Cys2Cys2-zinc-finger (18–62 aa) domains (*Fig. 1*).

**Table 2:** Characteristics of the exon-intron structure of the YABBY3 gene in the examined tomato accessions

Species/subspecies/cultivar	NCBI number	Exon-intron structure of YABBY3													Total length, bp	cDNA, nt	Protein, aa residues
		Exon I	Intron I	Exon II	Intron II	Exon III	Intron III	Exon IV	Intron IV	Exon V	Intron V	Exon VI	Intron VI	Exon VII			
S. cheesmaniae	KY952537	102	546	150	223	127	314	49	374	76	425	75	180	72	2,713	651	216
S. galapagense	KY952538	102	531	150	222	127	316	49	373	76	426	75	180	72	2,699	651	216
S. lycopersicum cv. Heinz ^*^	ID:101247051	102	536	150	222	127	316	49	373	76	426	75	179	72	2,703	651	216
S. lycopersicum var. humboldtii	KY952544	102	538	150	221	127	313	49	373	76	427	75	180	72	2,703	651	216
S. lycopersicum cv. Silvestre recordo	KY952543	102	537	150	222	127	316	49	372	76	426	75	180	72	2,704	651	216
S. pimpinellifolium var. racemigerum	KY952549	102	537	150	222	127	316	49	373	76	426	75	180	72	2,705	651	216
S. chmielewskii	KY952540	102	537	150	222	127	314	49	371	76	426	75	180	72	2,701	651	216
S. neorickii	KY952545	102	469	153	218	127	313	49	376	76	426	75	166	72	2,622	654	217
S. arcanum	KY952547	111	530	150	228	127	316	49	374	76	426	75	166	72	2,700	660	219
S. chilense	KY952539	102	533	150	224	127	311	49	374	76	426	75	165	72	2,684	651	216
S. corneliomulleri	KY952541	111	535	150	227	127	314	49	361	76	425	75	166	72	2,688	660	219
S. habrochaites	KY952542	102	545	150	222	127	274	49	361	76	421	75	178	72	2,652	651	216
S. pennellii ^*^	ID:107026918	102	522	144	222	127	312	49	372	76	414	75	179	72	2,666	645	214
S. peruvianum	KY952546	111	539	150	228	127	316	49	365	76	427	75	169	72	2,704	660	219
S. peruvianum var. dentatum	KY952548	111	485	150	221	127	312	49	374	76	426	75	180	72	2,658	660	219
S. tuberosum ^*^	ID:102577797	114	552	147	271	127	314	49	398	76	417	75	175	72	2,787	660	219
A. thaliana ^*^	ID:827914	102	97	138	101	151	93	49	119	76	136	99	440	81	1,682	696	231

^*^Sequences from the NCBI database.

In the 9 analyzed accessions, including all red-fruited and three green-fruited *(S. chmielewskii, S. chilense, *and *S. habrochaites) *species, *YABBY3 *cDNA was 651 bp (*Table 2*). In *S. neorickii, *cDNA was 654 bp due to TCA duplication in the second exon (N66_H67insH in amino acid sequence). In *S. arcanum, S. corneliomulleri*, *S. peruvianum, *and *S. peruvianum *var. *dentatum, *it was 660 bp due to 9 bp insertion in the first exon (P17_S18insPPP). In *S. pennellii*, which is known to be the most ancient species [32], cDNA of 645 bp was due to 6 bp deletion in the second exon (H67del, H68del). Accordingly, the length of the YABBY3 orthologs was 217 aa *(S. neorickii), *219 aa *(S. arcanum, S. corneliomulleri, S. peruvianum*, and *S. peruvianum *var. *dentatum), *and 216 aa (other accessions). Interestingly, among the previously described conserved YABBY1/3-characteristic motifs, *Solanum *YABBY3 orthologs included the clade-specific motifs FIL-A, -D, -E, and -G, but no FIL-B and -C, which are usually localized in the inter-domain region [12] (*Fig. 1*).

**Table 3:** The ANOVA analysis of YABBY3 gene expression in tomato species using Welch’s t-test.

S. lycopersicum cv. Silvestre recordo			
	Leaf	Bud	Flower
Bud	0.0012		
Flower	0.6189	0.0007	
Fruit	< 0.0001	< 0.0001	< 0.0001
S. chmielewskii			
	Leaf	Bud	Flower
Bud	0.0242		
Flower	0.1117	0.5025	
Fruit	< 0.0001	< 0.0001	< 0.0001
S. peruvianum var. dentatum			
	Leaf	Bud	Flower
Bud	< 0.0001		
Flower	0.1014	< 0.0001	
Fruit	< 0.0001	0.3049	< 0.0001
S. habrochaites			
	Leaf	Bud	Flower
Bud	< 0.0001		
Flower	< 0.0001	< 0.0001	
Fruit	< 0.0001	< 0.0001	< 0.0001

^*^p-values < 0.05 are considered as significant.

When compared with the previously characterized *S. lycopersicum *cv. Heinz *YABBY3 *(ID: 101247051), in the *YABBY3 *genes of the analyzed accessions, 317 variable sites, mostly localized in introns, were revealed. In the exons, 24 substitutions were detected, and 8 of them were nonsynonymous. Substitutions detected in cDNA were localized mainly in the sequence encoding the inter-domain region and at the 3’-terminus. In the region encoding the zinc-finger domain, only one substitution was detected: A59G transition in *S. galapagense*, which leads to a glutamine on arginine substitution, Q20R (*Fig. 1*). The sequence encoding the YABBY-domain revealed five nucleotide substitutions, and only one of them, A434G transition in *S. peruvianum *var. *dentatum *(3966), leads to a glutamic acid on glycine substitution, E145G (*Fig. 1*).

In YABBY3 proteins, 4 out of 11 aa substitutions (S64C, Y76C, D116G, and E145G) (*Fig. 1*) are considered to be radical (physicochemical distance according to Grantham’s matrix < 57.9). At the same time, an assessment using PROVEAN, generalizing known algorithms for a charge of aa substitutions and indels, revealed only one radical substitution (E145G in the *S. peruvianum *var. *dentatum *YABBY-domain*), *whereas the other substitutions, deletions, and insertions were rated as neutral. The possible effect of substitutions on the protein function needs further experimental analysis.

**Fig. 2:**
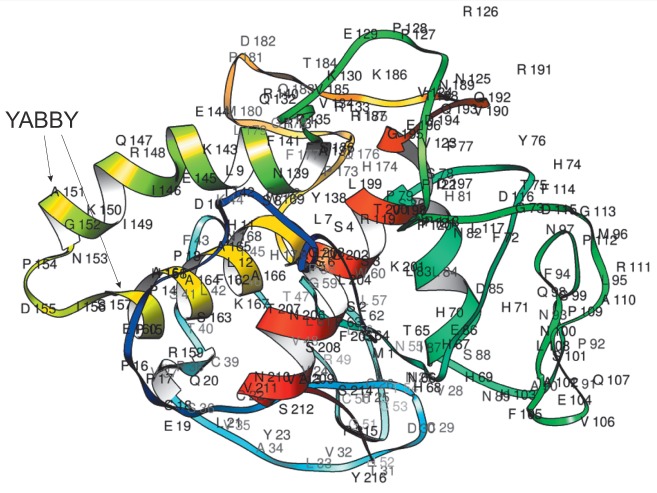
*S. lycopersicum *cv. Heinz YABBY3 (XM_004245689) tertiary structure (Phyre2): α-helices forming the YABBY domain are indicated by arrows

Modeling (Phyre2) of the YABBY3 three-dimensional structures showed a disordered organization of more than 60% of the sequence, while 29% were predicted with a confidence of more than 90% based on the known HMG-like protein structures (PDB: d1qrva, d1k99a etc.). The reliably predicted sequence was represented by a HMG-like YABBY domain [10] consisting of two α-helices connected by a loop (helix-loop-helix) (*Fig. 2*). The HMG-domain presumably binds to the DNA minor groove and bends the double helix at that point [38].

**Fig. 3:**
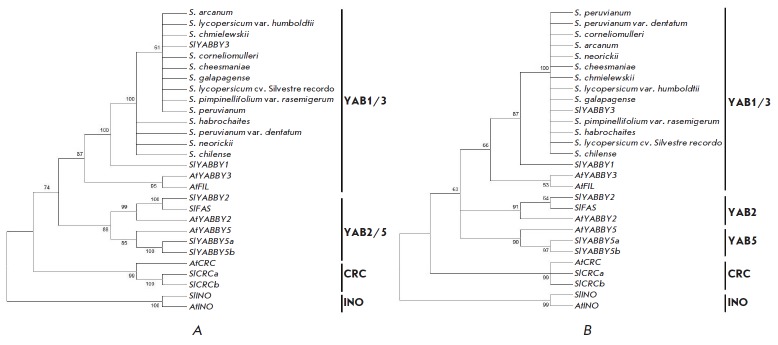
Phylogeny of *YABBY *genes in *S. lycopersicum *(Sl) and *A. thaliana *(At) based on cDNA (A) and amino acid sequences (B) (MEGA7.0, ML method; (A) – Hasegawa-Kishino-Yano model [39]+ Gamma distributed with invariant sites), (B) – Dayhoff model [40]+ Gamma distributed)

**Fig. 4:**
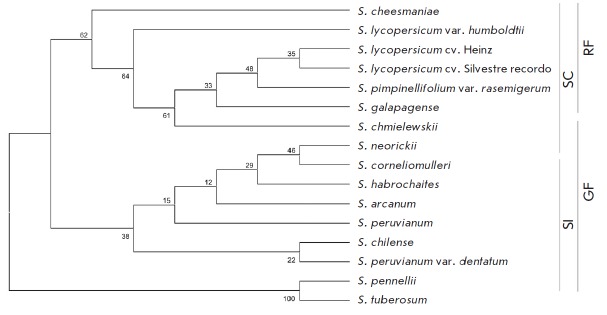
Phylogenetic tree based on *YABBY3 *genomic sequences of the accessions of cultivated and wild tomato species and rooted with *S. tuberosum YABBY3 *(MEGA7.0, ML method, model HKY + G+I). RF – red-fruited accessions; GF – green-fruited accessions; SC – self-compatible accessions; SI – self-incompatible accessions

The phylogenetic analysis showed that all known *S. lycopersicum YABBY *genes are clustered with the corresponding *A. thaliana *orthologs (*Fig. 3*). On the cDNA-based dendrogram, *YABBY* genes formed four sub-clusters: YAB1/3 (*YABBY1- *and *YABBY3-*like genes*); *YAB2/5 (*YABBY2*-, *YABBY5-, *and *FAS-*like genes*); *CRC (*CRC-*like genes); and INO (*INO*-like genes) (*Fig. 3A*). The clusters resulting from the analysis of the amino acid sequences (*Fig. 3B*) were similar to those described above, except for YABBY2 and YABBY5, which formed separate sub-clusters corresponding to the previously proposed classification of the YABBY family into five subfamilies [10, 23]. The phylogenetic analysis based on the *YABBY3 *genomic sequences clustered the analyzed tomato accessions into two groups with a branch of the most ancient *S. pennellii* and potato *S. tuberosum* (*Fig. 4*). The results generally agreed with the tomato division into green-fruited and red-fruited, as well as self-compatible and self-incompatible, groups. At the same time, two self-compatible green-fruited species, *S. chmielewskii *and* S. neorickii, *fell into opposing clusters, which apparently corresponds to an evolutionary boundary point where red-fruited self-compatible species originated from green-fruited self-incompatible ones.

**Fig. 5:**
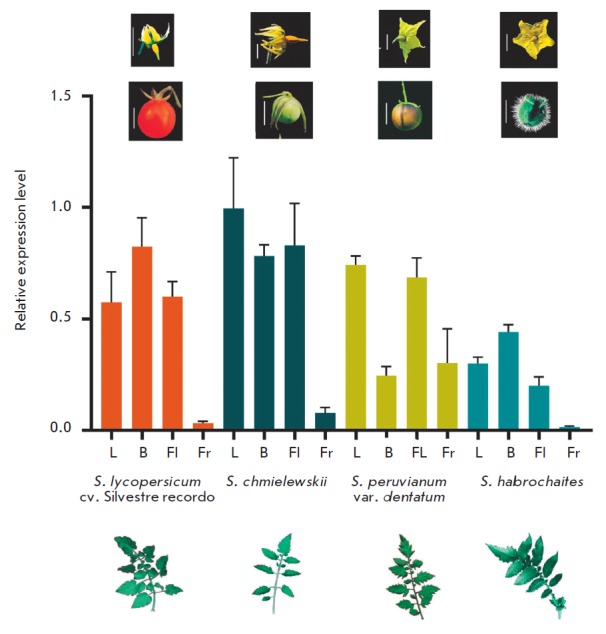
Relative *YABBY3* expression in the leaves (L), young buds (B), opened flowers (Fl), and green immature fruits (Fr) of four tomato accessions

The *YABBY *genes expression in angiosperms suggests that *YABBY1*/*3 *genes preserved their ancient expression pattern [12], transcribing in the abaxial portion of the primordia of all aboveground lateral organs (except for ovules) [25, 41]. This is confirmed by our data on *YABBY3* expression in the vegetative and reproductive organs of *S. chmielewskii*, *S. lycopersicum* cv. Silvestre recordo, *S. habrochaites*, and *S. peruvianum* var. *dentatum*. In *S. habrochaites, *gene expression in leaves is somewhat higher than that in flowers, while the other three species have no statistically significant differences in *YABBY3 *expression levels in leaves and flowers (*Fig. 5*, *Table 3*). At the same time, almost no *YABBY3 *expression was detected in the fruits of the studied species, except for *S. peruvianum *var. dentatum (*Fig. 5*). These four species were selected for expression analysis, since they belong to four groups that are evolutionarily distant from each other. *S. lycopersicum* is a red-fruited, self-compatible species of relatively recent origin; *S. chmielewskii* is a green-fruited, but self-compatible, species, and its position on the evolutionary tree is between red-fruited self-compatible and green-fruited self-incompatible species; *S. peruvianum* is a representative of the green-fruited self-incompatible species; and, finally, *S. habrochaites* (green-fruited, self-incompatible) is considered as one of the most ancient tomato species [32]. The *YABBY3 *expression pattern in *S. peruvianum *var. *dentatum *is somewhat different from that in other analyzed accessions, although the reason for the low-level expression in buds is not fully understood (*Fig. 5*). In the analyzed organs of *S. habrochaites*, the *YABBY3* expression dynamics is similar, but the transcription level is almost twice lower than that in *S. lycopersicum *and *S. chmielewskii*. In general, the identified *YABBY3 *expression patterns in *S. lycopersicum*, *S. chmielewskii, *and *S. habrochaites *were similar to those in *S. pimpinellifolium*, wherein the *YABBY3 *expression level is maximal in young buds and decreases along with flower-to-fruit development [30].

It has been shown that, in *A. thaliana*, both the *YABBY3 *constitutive expression and its knockout lead to an abnormal development of leaves and flowers due to the lack of polar differentiation in the organs [18]. The variability of this gene expression level can also affect the organ structure and morphophysiology; in particular, the leaves, flowers, and fruits of the analyzed tomato accessions. Significant levels of gene expression in *S. peruvianum* var. *dentatum* fruits may be indicative of a possible preservation of abaxial tissue identity in the fruit skin.

## CONCLUSION

In this study, *YABBY3-*orthologous genes were detected in 13 accessions of cultivated and wild tomato species. These genes encode transcription factors that play a key role in determining the abaxial-adaxial asymmetry of all aboveground plant lateral organs. The structure of *YABBY3 *genes and the encoded proteins is similar to that of the previously characterized members of the YABBY family. The phylogenetic and expression analysis confirmed that the identified genes belong to the *YABBY1*/*3* subfamily and may have conserved functions in different tomato species.

## Abbreviations

CRC: CRABS CLAW
INO: INNER NO OUTER
FIL: FILAMENTOUS FLOWER
qRT-PCR: quantitative real-time PCR
